# SynAsk: unleashing the power of large language models in organic synthesis†

**DOI:** 10.1039/d4sc04757e

**Published:** 2024-11-18

**Authors:** Chonghuan Zhang, Qianghua Lin, Biwei Zhu, Haopeng Yang, Xiao Lian, Hao Deng, Jiajun Zheng, Kuangbiao Liao

**Affiliations:** a Guangzhou National Laboratory Guangzhou Guangdong 510005 PR China liao_kuangbiao@gzlab.ac.cn; b AIChemEco Inc. Guangzhou Guangdong 510005 PR China

## Abstract

The field of natural language processing (NLP) has witnessed a transformative shift with the emergence of large language models (LLMs), revolutionizing various language tasks and applications, and the integration of LLMs into specialized domains enhances their capabilities for domain-specific applications. Notably, NLP has made significant strides in organic chemistry, particularly in predicting synthetic tasks, paving the way for the development of LLMs tailored to the organic chemistry field. In this work, we introduce SynAsk, a comprehensive organic chemistry domain-specific LLM platform developed by AIChemEco Inc. By fine-tuning an LLM with domain-specific data and integrating it with a chain of thought approach, SynAsk seamlessly accesses our knowledge base and advanced chemistry tools in a question-and-answer format. This includes functionalities such as a basic chemistry knowledge base, molecular information retrieval, reaction performance prediction, retrosynthesis prediction, chemical literature acquisition, and more. This novel methodology synergizes fine-tuning techniques with external resource integration, resulting in an organic chemistry-specific model poised to facilitate research and discovery in the field. Accessible at https://synask.aichemeco.com, SynAsk represents a significant advancement in leveraging NLP for synthetic applications.

## Abstract 2

SynAsk is a chemistry-specific LLM platform, fine-tuned with domain data and integrated with in-house tools. It supports tasks like reaction prediction, retrosynthesis, and literature retrieval, advancing organic synthesis research.

## Graphical abstract

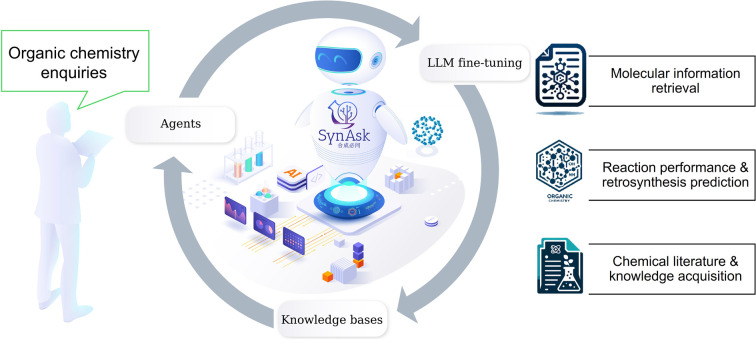

## 1 Introduction

In recent years, the field of natural language processing (NLP) has undergone a revolutionary shift with the emergence of large language models (LLMs), advanced artificial intelligence systems trained on massive datasets to understand and generate human-like text across various language tasks and applications. At the core of LLMs lies the remarkable technology of generative pre-trained transformers (GPT).^1^ Developed by OpenAI, GPT models like ChatGPT^2^ have gained widespread attention and adoption for their capacity to produce coherent and contextually relevant text. ChatGPT, in particular, represents a milestone in conversational AI, enabling human-like interactions that go beyond scripted responses. Evolving from ChatGPT to GPT-4 (ref. 3) through continual learning from vast datasets allows these models to grasp nuances of language and context, making them versatile tools for diverse tasks, from assisting in creative writing to generating videos. While GPT models have dominated the landscape, other models like Qwen^4^ and LLaMA^5^ also make significant contributions to the field, and these models are open-sourced for the community to utilize. Qwen, primarily trained from Mandarin Chinese language sources, is renowned for its robustness in question-answering tasks, leveraging a different architecture and training approach. On the other hand, LLaMA specializes in language understanding and inference tasks, offering unique capabilities in semantic analysis and knowledge extraction.

Beyond ChatGPT and other models, LLMs encompass a spectrum of applications across vertical domains. Domain-specific and customized data have been collected and labeled to fine-tune these LLMs. One of the key benefits of vertically specialized LLMs is their capacity to bolster domain-specific applications. By refining their expertise within a particular domain, these models possess the capability to delve deeply into the nuances of the subject matter, rendering them invaluable tools for professionals operating in specialized domains. For instance, a legally specialized LLM, namely DISC-LawLLM,^6^ can provide precise legal counsel, draft contracts, and facilitate intricate legal research, thereby streamlining processes and conserving resources for legal practitioners. Similarly, a medically specialized LLM, namely MultiMedQA,^7^ can assist physicians in diagnosing rare conditions, proposing tailored treatment plans, and staying updated on the latest technologies in medical research.

The integration of NLP into organic chemistry has brought about a revolution in research and discovery. Molecules and reactions can now be represented using SMILES (Simplified Molecular Input Line Entry System), a textual notation for depicting high-dimensional chemical structures.^8^ NLP techniques have been employed to tackle organic synthesis tasks using SMILES strings, treating the synthesis problem as a sequence generation task. This approach involves training machine learning models to predict the sequence of molecules and reactions necessary to synthesize a target molecule based on desired products. These models learn from extensive datasets of annotated reactions, where each reaction is represented as a sequence of SMILES strings. Leveraging the patterns and rules encoded in the data, these models can generate plausible synthesis pathways.^9,10^

LLMs have found applications in organic chemistry as well. However, without further tuning with organic chemistry domain-specific data, researchers have evaluated five LLMs in tasks related to organic chemistry, including reaction prediction and retrosynthesis. While these models provide reasonable results in classification or ranking tasks like yield prediction and reagent selection, they face challenges in generative tasks that require a deep understanding of molecular structures.^11^ This difficulty may stem from the highly experimental nature of organic chemistry, the lack of labeled data, and the limited scope and applicability of computational tools in this field.^12^ To bridge this gap and motivate further exploration of LLM potential in chemistry, several domain-specific LLMs for organic chemistry have been developed. ChemCrow^12^ was the first proposed LLM in chemistry aimed at enhancing its capabilities through external tools. It employs chain-of-thought (CoT) strategies,^13^ which are a series of intermediate reasoning steps to improve LLMs' ability to understand tasks from prompts. ChemCrow also utilizes LangChain,^14^ a framework to connect the LLM with multiple external tools downstream to solve specific tasks and return answers back to the LLM. However, this method relies on the reliability of tools, and general LLMs may not comprehensively understand prompts and link to the correct tools to solve specific tasks.Another approach, ChemLLM,^15^ was proposed to transform structured chemical data into forms suitable for LLMs to fine-tune the LLaMA model. ChemLLM excels in tasks such as cheminformatics programming. However, its performance may not be as robust as comprehensive models like ChatGPT-4, possibly due to human biases in the collection of incomplete structural chemical data.

**Fig. 1:**
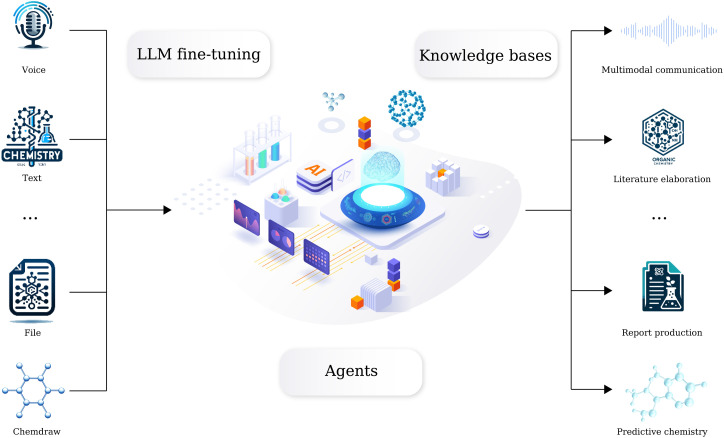
The overview of the SynAsk platform.

We have long been dedicated to AI in chemistry research, developing a series of machine learning and computational based tools to solve fundamental organic chemistry tasks. However, we recognize that directly connecting these tools to large language models (LLMs) may not yield appropriate results. Here we introduce a comprehensive domain-specific LLM for organic chemistry developed by AIChemEco, named SynAsk, as shown in Fig. 1. An LLM was refined using a limited set of domain-specific chemistry data and integrated with a chain-of-thought approach to understand user prompts. Our aim is to utilize LangChain to seamlessly connect SynAsk with our existing suite of tools, addressing specific user inquiries, drawing on the framework of LangChain-Chatchat.^16^ This methodology allows us to combine fine-tuning techniques with the integration of external resources, resulting in the development of an organic chemistry-specific model. This framework is adaptable, and with access to high-quality data from other domains, such as inorganic chemistry, materials science, and catalysis, SynAsk has the potential to extend its capabilities to these fields, broadening its impact across the chemical community. The model can be accessed at https://synask.aichemeco.com.

## 2 Methods

To construct the comprehensive model integration platform, our approach unfolds along three primary dimensions: utilizing a powerful foundation LLM as the base for SynAsk, crafting more effective prompts and implementing fine-tuning to the foundation model, and connecting with multiple tools to assemble a chemistry domain-specific model platform.

### 2.1 Selection of a foundation LLM

Through various experiments, we have recognized that for the foundation LLM to effectively understand prompts from end-users and apply insights to decide whether to provide LLM inference answers or use specific tools to resolve downstream tasks, it needs to have at least 14 billion parameters. Therefore, only foundation models with over 14 billion parameters were considered. The capabilities of the LLM were assessed using indicators such as Massive Multi-task Language Understanding (MMLU),^17^ Multi-level multi-discipline chinese evaluation (C-Eval),^18^ GSM8K,^19^ BIG-Bench-Hard (BBH)^20^ and Measuring massive multitask language understanding in Chinese (CMMLU),^21^ as elaborated in Section S1 of the ESI.† These indicators collectively offer a comprehensive assessment of a model's proficiency, covering areas such as linguistic understanding, mathematical reasoning, contextual comprehension, multi-modal integration, and the application of Chain-of-Thought (CoT), which evaluates the fluency of LLMs' integration with external tools. This evaluation framework underscores the essential and diverse skills a model must possess to adeptly address complex real-world problems.

As indicated in Table S1,†^4^ the Qwen series^4^ outperforms other models with equivalent parameter counts, including LLaMA2,^22^ ChatGLM2,^23^ InterLM,^24^ Baichuan2 (ref. 25) and Yi^26^ in these areas. Additionally, our testing has confirmed that the Qwen series is more compatible with our framework, especially with the release of Qwen-1.5, which provides us with more options. We acknowledge that the GPT series,^2^ particularly GPT-4,^3^ scores higher than Qwen. However, at the time of this work, GPT-4 has not been open-sourced and requires paid API tokens to use as a foundation model. To ensure SynAsk remains publicly accessible, we opted to use only open-sourced foundation LLMs and developed an architecture that allows for smooth switching of the foundation LLM, as discussed in Section 2.4.

### 2.2 Refinement to a more reasonable prompt

To improve the model's performance in two key areas—providing more targeted responses in the chemical domain and enhancing its ability to efficiently utilize tools—we refined our prompt templates through iterative testing and adjustments. We guide the model to generate responses that are not only accurate but also consistent with specific demand expectations. This process encourages the model to become more deeply involved in the task at hand, reducing ambiguity and focusing its attention. These optimized guidance models function as both competent chemists and skilled tool users, establishing a more focused, efficient, and effective interaction between the model and the user.

**Fig. 2:**
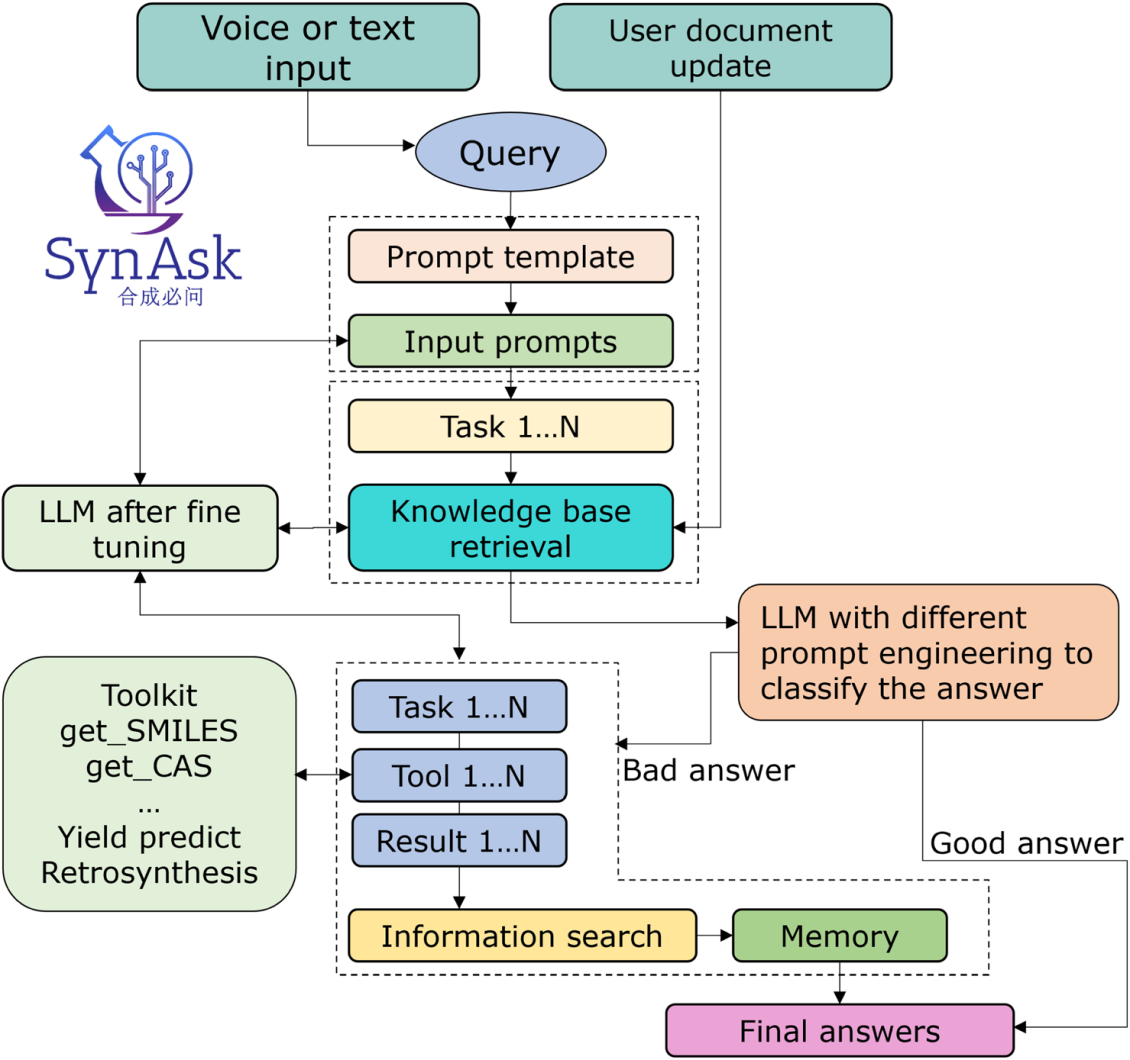
The workflow of the SynAsk platform: from the input to the final answer.

In our integrated platform, utilizing the classification function of LLMs is particularly crucial, as illustrated in Fig. 2. Since this platform extends from our existing NLP project, we believe it inherently possesses enhanced capabilities. To further train it, we employ a tailored hint project, where the model's role is set as a chemist evaluating and scoring the generated results. This project provides several examples to guide the model. This setup enables the model to discern whether responses augmented by the knowledge database meet the criteria, thereby classifying the results into those that meet expectations and those that do not.

### 2.3 Fine-tuning of the LLM

The selected model underwent fine-tuning to specialize it further in the field of chemistry, ensuring its engagement in professional chemical dialogues, particularly in organic synthesis. The fine-tuning process comprised two iterations, with data processed accordingly for each iteration.

• The first iteration was supervised fine-tuning: this stage focused on enhancing the model's cognitive abilities, reinforcing its identity as an expert in chemistry. The objective was to delve deeper into the model's capabilities within the chemistry domain without expanding its original data source. This approach allowed the model to utilize existing data more effectively to solve chemical problems.

• The second iteration was instruction-based fine-tuning: the aim here was to improve the model's reasoning and tool invocation capabilities, thereby enhancing its chain of thought. It learned to differentiate between various types of chemical identifiers, such as SMILES and CAS numbers, rather than treating them as ordinary words or sequences of numbers.

The rationale for dividing the fine-tuning into two stages is threefold:

• Clear and controllable training: each fine-tuning task addressed a specific sub-problem, ensuring clarity and controllability in the training process and outcomes. This approach facilitates adjustments and improvements based on the results of previous fine-tuning, gradually enhancing the model's performance on specific tasks.

• Prevention of interference: segregating the tasks prevents confusion and interference between them. Combining all tasks into a single fine-tuning session might lead to instability in training or reduced performance.

• Accelerated training: this approach speeds up the training process. By simplifying each fine-tuning task, the training becomes more efficient, yielding quicker results and feedback. The shorter training times for each task contribute to a faster overall training cycle.

After fine-tuning, detailed techniques, procedures, and the necessary equipment are elaborated in Section S2 of the ESI.† Post-fine-tuning, our emphasis mainly lies on the model's ability to demonstrate Chain of Thought (CoT) in its output. Following the fine-tuning process, we provide two examples of the model's simplified output format:
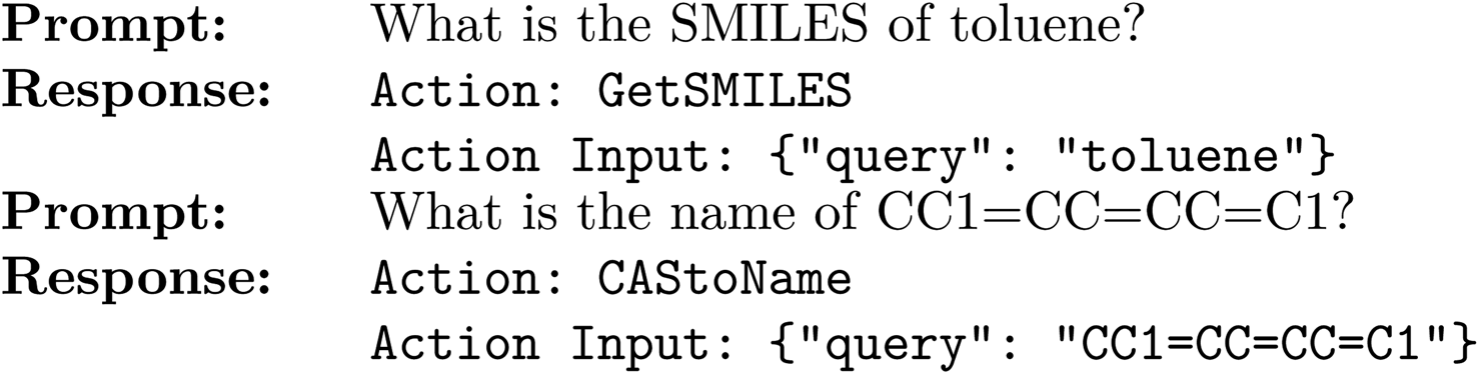


Notably, the power of these fine-tuned results is significantly enhanced when used in conjunction with appropriately designed prompting strategies and specially designed tool formats. These responses demonstrate the model's ability to identify the required action and its corresponding input from the prompts. However, within our framework, these responses are not the final outcome. Instead, they serve as intermediate prompts to be re-fed into the model. This intermediary step is pivotal, enabling the model to discern the specific tool it requires (*e.g.*, ‘GetSMILES’ for the initial example) and to process the “Action Input” (*e.g.*, query: ‘toluene’) utilizing the designated tool. Subsequently, the expansive model amalgamates the tool's output with its vast knowledge base, culminating in the generation of a final answer.

### 2.4 SynAsk architecture

In the final phase, we implemented the LangChain framework to seamlessly integrate our local knowledge base with both internal and external open-source tools and APIs. Its primary role is to interpret the outputs from the language models, converting them into a format understandable by external tools, thus facilitating the execution of corresponding actions. Simultaneously, it translates the responses from these tools back into a form comprehensible by the language models. Furthermore, LangChain's support for context management enables it to track the interaction history between users and the system. This enhances the system's ability to understand user intentions and maintain session continuity during interactions with external tools. Its scalability ensures that the system can adapt to technological advancements and changing user demands, providing a dynamic and responsive framework for our integration needs. The LangChain framework serves as a pivotal bridge, culminating in a logically coherent and systematically robust integration platform known as SynAsk.

The structural framework of SynAsk is illustrated in Fig. 2. Initially, it can accept both voice and text inputs as queries, which are then segmented into multiple tasks by an LLM and matched against our knowledge base. At this stage, users also have the option to upload their local files as supplementary knowledge or directly engage in conversations with the uploaded files. Once matching texts are obtained, the large model synthesizes the content along with its understanding of the question to deduce a conclusion, thereby generating a result. Subsequently, the model evaluates this result to determine if it meets the expected criteria. If the outcome is deemed satisfactory, it is directly outputted as the Final Answer. Conversely, if the results do not meet expectations, we will enter our customized Agent Q&A mode and call our tools to answer. Finally, the tool output is combined with the LLM's self-knowledge to generate the final answer.

In the SynAsk architecture, although we currently utilize Qwen-1.5 as the foundation LLM, we recognize the ongoing revolutions in LLM technology. Consequently, we have developed a workflow to swiftly adjust the foundation model and fine-tune the domain-specific data. This approach ensures that SynAsk can continuously update and iterate, leveraging the latest advancements in foundation LLMs.

### 2.5 SynAsk toolsets

Cheminformatics tools are seamlessly connected with SynAsk through LangChain to provide comprehensive organic synthesis answers. This includes a variety of machine learning-powered tools developed both internally and by external teams, all dedicated to solving organic synthesis tasks. At the time of publishing this work, 12 internal tools and 10 external tools have been integrated into SynAsk. External tools are appropriately cited with their origins. With the rapid development of this field, we anticipate an increasing influx of tools joining SynAsk. These tools are categorized into molecular, reaction tools, and others, with a number of advanced in-house tools elaborated in Section 2.5.5.

#### 2.5.1 Molecular information retrieval

This category encompasses tools designed for querying various molecular identifiers and properties. Functions include retrieving Chemical Abstracts Service (CAS) numbers, Simplified Molecular Input Line Entry System (SMILES) strings, molecular weights, assessing molecular similarity, identifying types of functional groups, and checking the regulatory status of molecules. The respective tools for these purposes are:

• 
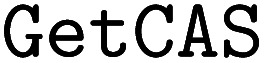
 – for CAS number retrieval^27^

• 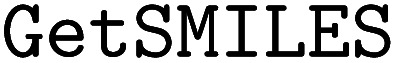 – for obtaining SMILES strings^27^

• 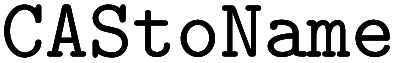 – to convert CAS numbers to chemical names^28^

• 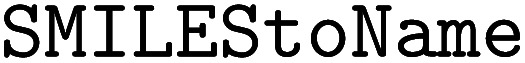 – to convert SMILES strings to chemical names^28^

• 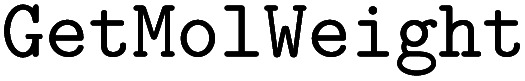 – for calculating molecular weights

• 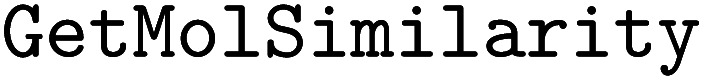 – to determine molecular similarity

• 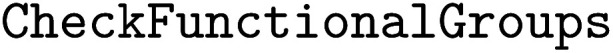 – for functional group identification

• 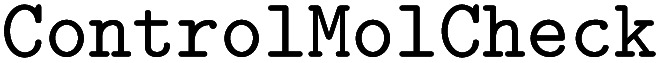 – to check if a molecule is controlled.

#### 2.5.2 Reaction performance and retrosynthesis prediction

This category aids in querying chemical reaction conditions, planning chemical reaction pathways, predicting chemical reaction yields, performing retrosynthetic analysis, and predicting reaction derivatives. Tools provided for these functions include:

• 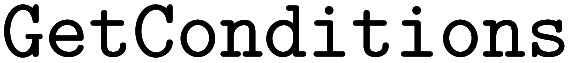 – to query chemical reaction conditions

• 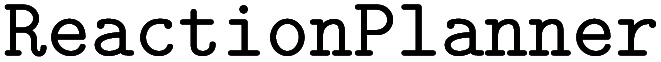 – for planning chemical reaction pathways^29^

• 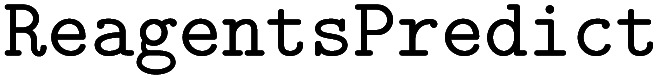 – to predict reagents in chemical reactions

• 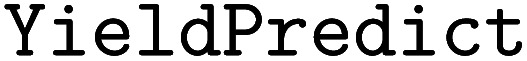 – for predicting chemical reaction yields

• 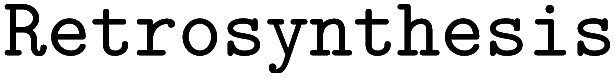 – to perform retrosynthetic analysis

• 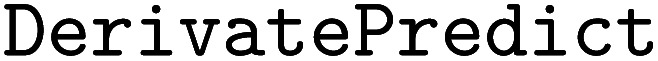 – to predicts the derivatives from a chemical reaction, using reactants' names or SMILES, enhancing the exploration of reaction outcomes.

• 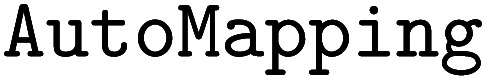 – to identify the position of each atom in the molecules before and after a chemical reaction.^30,31^

#### 2.5.3 Chemical literature and knowledge acquisition

Dedicated to acquiring chemical literature and extracting chemical knowledge, tools in this section include:

• 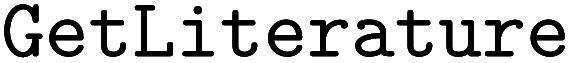 – for retrieving literature^32,33^

• 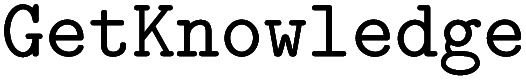 – to obtain chemical knowledge^33^

• 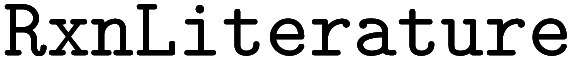 – for sourcing reaction-specific literature.

#### 2.5.4 Miscellaneous

This section covers a diverse array of functions including drawing chemical molecular structures and balancing chemical equations. Tools include:

• 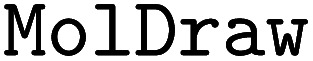 – for drawing chemical molecular structures

• 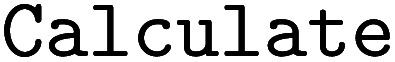 – a general-purpose calculation tool

• 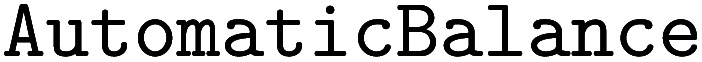 – to automatically balance chemical equations^34^

• 
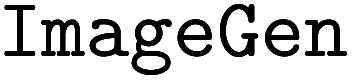
 – for generating and searching images.^33^

#### 2.5.5 Advanced in-house analytical tools

##### 2.5.5.1 YieldPredict

**Fig. 3:**
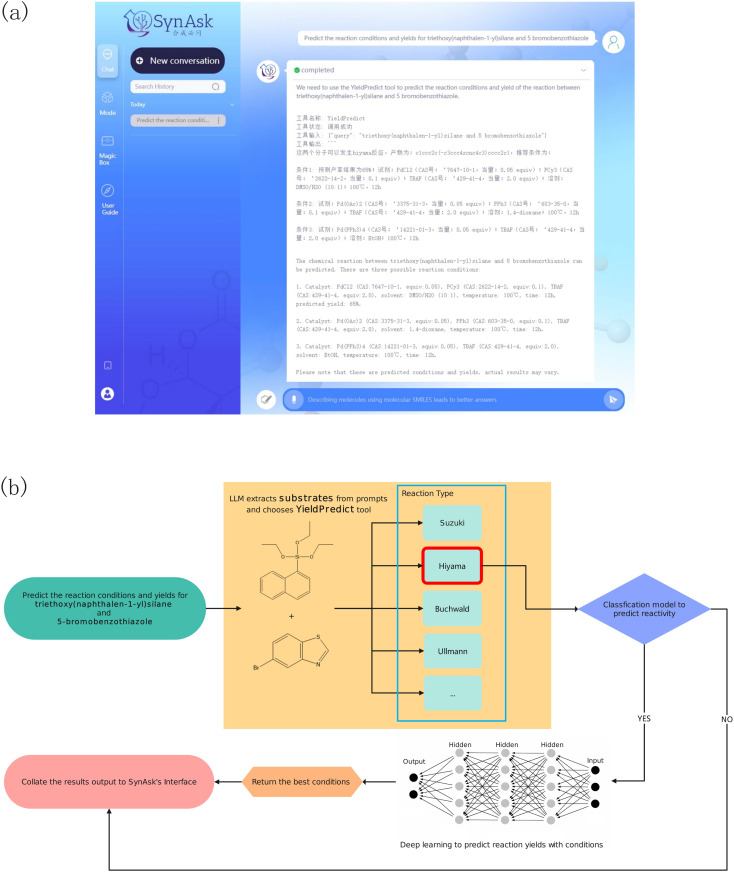
An example of the YieldPredict tool workflow for predicting the reaction yield of triethoxy(naphthalen-1-yl)silane and 5-bromobenzothiazole: (a) the user interface of SynAsk, (b) the thinking process of the YieldPredict tool.

This is an API tool linked with our self-developed reaction yield prediction tool. By inputting at least two substrates, either in their molecular name or molecular SMILES, this tool can identify the possible reaction types of the molecules by querying our reaction template library. With the known reaction types, the molecules are passed into the reaction models as substrates. The models then suggest products and the most suitable reaction reagents and conditions for the substrates. For example, upon asking the reaction yield of triethoxy(naphthalen-1-yl)silane and 5-bromobenzothiazole, the tool first parses the two molecules into the reaction templates as substrates (Fig. 3). This suggests Hiyama cross-coupling reactions. The two substrates are then parsed into the Hiyama reaction models, generating products and possible reaction yields under specific reaction reagents and conditions.

We have dedicated our efforts to developing data-driven reaction yield prediction models for common reaction types.^35–38^ For each model of a specific reaction type, we conduct chemical reaction experiments using high-throughput experimentation (HTE) techniques with various substrates. We developed a novel method designed to generalize the HTE substrate sampling and mimic real-world scenarios and generalize to more complex reactions across a broader chemical landscape. This approach involves projecting literature and patented reactions into chemical space, followed by virtual compound generation, dimensionality reduction, clustering, virtual compound filtering, and stratified sampling.^39^ This enables us to draw insights from existing literature data and identify areas where experimental data collection is necessary to augment an equitable data space for refining model training, thus facilitating more robust interpolation. We develop reaction models using machine learning techniques such as support vector machine (SVM) and NLP deep learning models like BERT (Bidirectional Encoder Representations from Transformers).^40^ These models are validated using external literature test data, achieving reasonable Mean Absolute Error (MAE), commonly below 0.15. As of the publication of this work, we have included 18 reaction types in this tool.

##### 2.5.5.2 GetConditions

This tool is a simplified version of YieldPredict. Instead of predicting the reaction product and yield, it provides rapid responses and suggests only the suitable reaction conditions and reagents for the substrates.

##### 2.5.5.3 Retrosynthesis

By inputting the desired target products, this tool generates numerous reaction pathways of molecules starting from buyable precursors. We have developed our own retrosynthesis model for this purpose. For a desired product, it is parsed into the reaction template library to find possible substrates and, consequently, the suitable reaction site for bond breakage. A reinforcement learning-trained agent selects the most suitable reaction from the candidates based on the forecasted synthesis difficulty and predicted reaction yield of the substrates (desired products at the previous step). This process is conducted recursively until the last substrates are buyable. At the output, we present the results in both textual form and as retrosynthetic route images. The algorithm of our retrosynthesis model will be published elsewhere.

### 2.6 Dynamic learning and knowledge base updates

To ensure SynAsk remains up-to-date with the latest developments in synthetic chemistry, our platform incorporates two mechanisms for dynamic learning and real-time knowledge base updates:

• Real-time knowledge base updates: SynAsk's knowledge base is designed to be updated in real-time. New tools and datasets can be integrated seamlessly by uploading the processed knowledge base or attaching new tools to the system. Once this is done, the platform team can implement a one-click update to make the newly integrated data or tools available for use by the model. This allows SynAsk to immediately leverage the latest experimental findings, tools, and databases in its predictions.

• Incremental learning framework: while SynAsk can dynamically update its knowledge base, the model also supports periodic updates through pretraining and fine-tuning. After collecting and processing new high-quality data, the model undergoes further pretraining to incorporate the new information. This step follows an incremental learning framework,^41,42^ which allows SynAsk to integrate new data without retraining the entire model from scratch. After sufficient testing, the updated model is deployed for real-time use.

Together, these mechanisms ensure that SynAsk can adapt to new discoveries and data, maintaining its relevance in the fast-evolving field of synthetic chemistry.

### 2.7 Ethical safeguards and risk management

To prevent misuse of SynAsk in ethically sensitive areas, we have implemented several protective measures. Ethical risks, such as using the platform to design illegal substances (*e.g.*, recreational drugs), create harmful chemicals, or develop environmentally dangerous compounds, are addressed through a comprehensive risk management framework. This framework integrates user accountability, preemptive model safeguards, and clear ethical guidelines to ensure responsible application of SynAsk in scientific research.

• User monitoring and model safeguards: SynAsk uses advanced natural language processing (NLP) techniques to monitor and categorize user queries in real-time. If the system detects queries related to sensitive or illegal topics—such as the synthesis of recreational drugs or hazardous substances—it automatically flags these interactions. In such cases, the platform provides cautionary warnings instead of detailed responses, and users may face temporary or permanent restrictions based on the severity and frequency of such queries. Additionally, during model pre-training and fine-tuning, we implemented safeguards to ensure that SynAsk cannot be used to generate potentially harmful or illegal compounds. Our knowledge base and integrated tools have been rigorously curated to exclude high-risk content, and new external data is continuously screened to prevent inappropriate use of the platform.

• Embedded ethical guidelines: SynAsk is governed by a clear set of ethical principles communicated to all users. These guidelines emphasize the importance of responsible and ethical research practices, particularly in fields with significant societal and environmental implications. By promoting transparency and responsibility, SynAsk aims to support scientific advancements while adhering to global ethical standards.

## 3 SynAsk performance

We evaluate the performance of SynAsk from two perspectives: its general ability as a large language model (LLM), and its proficiency in synthetic chemistry. Additionally, we provide several examples of SynAsk's outputs to demonstrate the platform's comprehension capabilities.

### 3.1 General ability of SynAsk

**Fig. 4:**
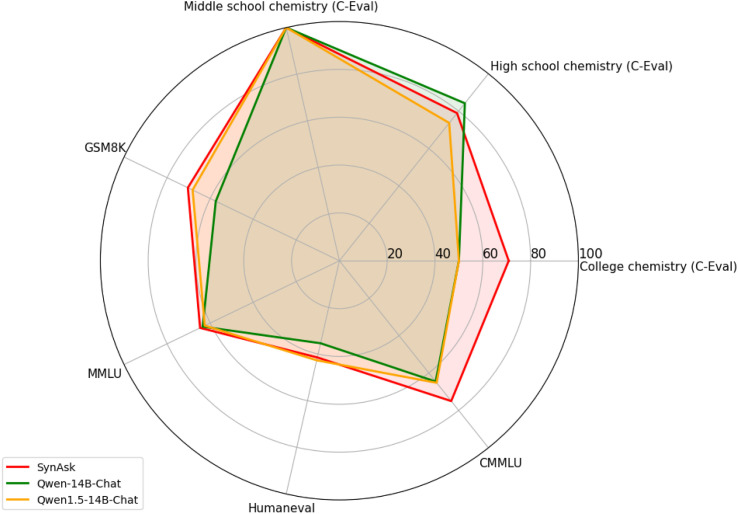
The comparison of the general ability between SynAsk and Qwen in seven aspects, including their applications in chemistry.

We evaluate the performance enhancements achieved through our first fine-tuning method on the SynAsk model based on OpenCompass,^43^ which serves as a universal evaluation platform for foundation LLMs. The efficacy of the method is demonstrated by its superior scores across various assessment indicators, particularly in its application to chemistry. The definitions of the general indicators used in Fig. 4 are provided in Section S1 of the ESI,† while the chemistry-related indicators are outlined in Section S3† along with examples. It's noteworthy that indicators such as College Chemistry, High School Chemistry, and Middle School Chemistry in the figure all stem from C-Eval. SynAsk significantly outperforms its foundation model predecessors. For example, in the area of College Chemistry, SynAsk achieves a remarkable score of 70.83%, compared to 50% by both Qwen-14B-Chat and Qwen1.5-14B-Chat. This signifies a substantial improvement, highlighting the model's enhanced ability to effectively utilize existing data sources for solving complex chemical problems.

Furthermore, the scores in other key benchmarks such as MMLU, GSM8K and CMMLU also reflect the overall enhancement of the SynAsk model. In CMMLU, which assesses cross-model multitask learning, SynAsk scored 75.03%, indicating its proficiency in integrating textual and visual information, crucial for multi-model applications. Similarly, its performance in MMLU and GSM8K benchmarks demonstrates its improved global knowledge comprehension and multi-step mathematical reasoning, respectively.

The advancements in SynAsk are attributed to the fine-tuning approach that leverages existing data sources more efficiently, thus enhancing the model's ability to address nuanced chemical contexts and complex reasoning tasks. This is particularly crucial for applications requiring deep understanding and contextual awareness, as indicated by the improvements in C-Eval scores.

These results collectively underscore the effectiveness of our fine-tuning methodology, confirming its potential to significantly boost performance across diverse linguistic and cognitive challenges, thereby reinforcing the model's utility in academic and practical applications.

### 3.2 Proficiency in synthetic chemistry

The primary proficiency of SynAsk in synthetic chemistry lies in its ability to predict reaction performance, such as the reaction yield, and to conduct retrosynthetic planning of target molecules, utilizing the embedded tools within SynAsk. Several case studies are presented and compared with benchmarks to evaluate the model's performance. While SynAsk has demonstrated strong performance in synthetic chemistry tasks, its architecture, which integrates fine-tuning and high-quality external tools, can be easily expanded to other domains. With the availability of reliable data and tools, this workflow could be adapted to areas such as inorganic chemistry, materials science, and catalysis, offering valuable insights and predictions in those fields as well.

#### 3.2.1 Reaction yield prediction

A number of reaction yield prediction models have been developed and widely used to forecast the performance of reactions for frequently encountered reaction classes. For instance, Doyle *et al.*'s palladium-catalysed Buchwald–Hartwig cross-coupling reaction model^44^ and Richardson *et al.*'s Suzuki–Miyaura cross-coupling reaction model^45^ are among the notable examples. These models were trained using self-developed high-throughput experimentation (HTE) reaction data employing machine learning algorithms. Schwaller *et al.*^46^ further enhanced the performance of these models using the same datasets through a pre-trained BERT model. While these methods effectively predict the product yield within the self-developed HTE reaction dataset, their applicability to predicting the product yield of external literature recorded reactions may be limited.

**Fig. 5:**
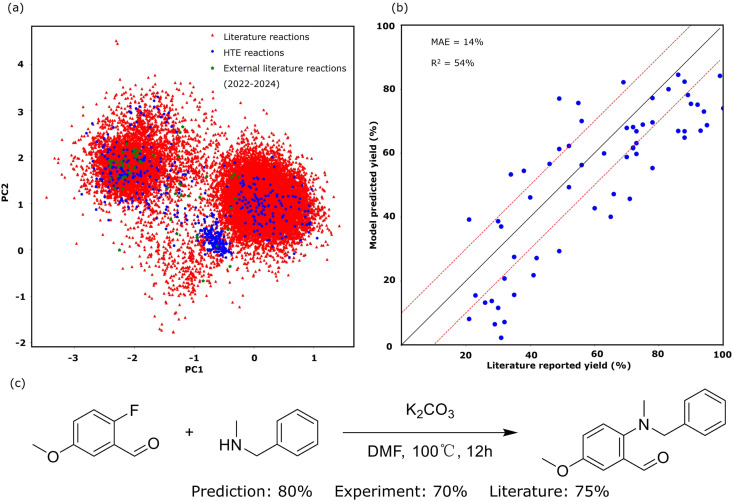
The S_N_Ar reaction model results: (a) the chemical space of S_N_Ar reactions under the HTE and literature recorded datasets, (b) the predicted yield *versus* experimental yield of the test dataset from the three different models, and (c) an example of the S_N_Ar reaction: *N*-methyl-1-phenylmethanamine reacting with 2-fluoro-5-methoxybenzaldehyde.

We tested our in-house nucleophilic aromatic substitution (S_N_Ar) reaction model embedded in SynAsk with both a test set and external literature reaction data. We performed five-fold cross-validation on the test set, which comprises unseen HTE reaction data, yielding a mean absolute error (MAE) of 11.7%. For the external literature reaction data, to minimize bias, we randomly collected 60 recently published S_N_Ar reactions from the last three years (2022–2024), including new substrate molecules never seen by the reaction model. The comparison between the model-predicted yield and literature-reported yield is presented in Fig. 5b, yielding an MAE of 14.1%. These recent published reactions encompass seven different reaction conditions. For example, *N*-methyl-1-phenylmethanamine reacting with 2-fluoro-5-methoxybenzaldehyde under K_2_CO_3_ and DMF is illustrated in Fig. 5c. The literature-reported yield of the product 2-(benzyl(methyl)amino)-5-methoxybenzaldehyde is 75%,^47^ whilst our model predicts 80% and our HTE experimental yield is 70%.

The decay in prediction accuracy observed when transitioning from HTE reactions to literature-reported reactions is primarily attributed to the increased complexity of substrates in literature reactions. These substrates are often more intricate and unseen by the model, thereby encompassing a wider range within the chemical space, as depicted in Fig. 5a. To compute the chemical space, we digitized the reactions using RXNFP pretrained reaction fingerprints^48^ and reduced them into two dimensions. Fig. 5a also weakly show three clusters of the S_N_Ar reaction. The slightly higher MAE of 14.1% in the external literature validation reflects the real-world scenario where the model is applied to predict the outcomes of experiments with greater substrate complexity and a wider range of reaction conditions. This external validation simulates the use of our in-house S_N_Ar model in practical, real-world applications, where it is expected to handle diverse and unseen situations. An MAE of 14.1% remains highly acceptable for yield prediction, as it enables chemists to reliably distinguish between high, medium, and low yields. This level of accuracy is particularly valuable for optimizing reaction conditions efficiently, helping chemists to prioritize promising experimental setups. This is particularly valuable for the interest of synthetic chemists.

In addition, we have included plots in Section S4 of the ESI† that compare our experimental validations with the model predictions for test datasets across four major reaction models: S_N_Ar reaction, Suzuki coupling reaction, Buchwald–Hartwig coupling reaction, and amide coupling reaction, further demonstrating the accuracy and reliability of our models across a diverse range of reaction types.

#### 3.2.2 Retrosynthetic route planning

We tasked SynAsk with planning retrosynthetic routes for 11 549 small molecule drugs recorded in the ChEMBL database.^49^ SynAsk successfully predicted retrosynthetic routes for 6358 molecules, suggesting step-by-step routes starting from buyable precursors. This accounts for 55% of the queried molecules. In contrast, a State-of-the-Art (SOTA) open-sourced retrosynthetic planning tool, AIZynthFinder,^50^ only suggested 3118 retrosynthetic routes, covering 27% of the queried molecules. This significant improvement highlights SynAsk's capability in retrosynthetic prediction, particularly for complex molecules where traditional methods may struggle.

**Fig. 6:**
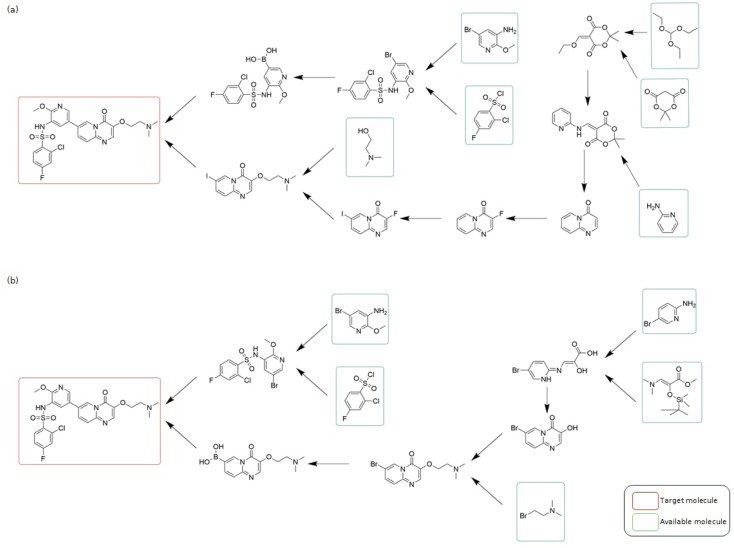
The comparison among synthetic routes of the target molecule Gilmelisib: planned by (a) SynAsk's retrosynthetic tool and (b) an experienced synthetic chemist.

As a case study, consider the retrosynthesis of Gilmelisib, a novel small molecule under investigation as a selective inhibitor of PIK3Cα, potentially treating cancers characterized by PIK3Cα mutations. SynAsk proposes a seven-step synthetic route with four precursors (as shown in Fig. 6a). This route matches the one suggested by an experienced human chemist in terms of length and number of precursors (as shown in Fig. 6b). SynAsk selects inexpensive precursors and employs common reactions such as Knoevenagel condensation and addition–elimination, which are well-aligned with established chemical knowledge. For cyclization, SynAsk offers a simpler reaction that was well published.^51^ Subsequently, halogenation can be easily realized using the *N*-halosuccinimide reagent. In contrast, AIZynthFinder did not propose any route for the target molecule, even after enriching its starting materials with our lists of buyable precursors. Additional synthetic routes for small molecule drugs are detailed in Section S5 of the ESI.†

While it is not our intention to claim that SynAsk surpasses human expertise or reaches human-level intelligence in retrosynthesis—such a conclusion would require Turing test-like evaluations^52,53^ or experimental validation—SynAsk's ability to generate plausible and efficient synthetic pathways demonstrates its value in assisting synthetic chemists with planning complex syntheses. Furthermore, this study underscores the importance of integrating machine learning techniques to complement traditional retrosynthesis methods.

### 3.3 Examples of the SynAsk platform outputs *versus* other LLMs

Here we present a comparative analysis of the performance of three LLMs – SynAsk, ChatGPT-4.0, and ChemCrow – in addressing synthetic chemistry queries. We evaluated their capabilities by inputting a set of synthetic questions, encompassing both general and professional inquiries, to assess their aptitude in providing accurate and relevant responses.

#### 3.3.1 General inquiries

Queries such as “Can you recommend me some reaction conditions for Suzuki cross-coupling?” or “Please help me find some literature related to C–H activation” were presented to all three LLMs. Across the board, each model exhibited proficiency in generating appropriate responses, showcasing their utility in aiding chemists with routine inquiries (details in Section S6 of the ESI†).

#### 3.3.2 Professional synthetic questions

A more rigorous evaluation was conducted by inputting a specific synthetic question: “tell me what reaction can occur between Nc1ccc2nccnc2c1.O=C(O)Cc1cc(F)cc(F)c1 and what the product is”. Here “Nc1ccc2nccnc2c1.O=C(O)Cc1cc(F)cc(F)c1” represents the SMILES syntax for quinoxalin-6-amine and 3,5-Difluorophenylacetic acid as substrates. The deliberate use of SMILES allows us to assess the LLMs' ability to recognize molecules from SMILES.

**Fig. 7:**
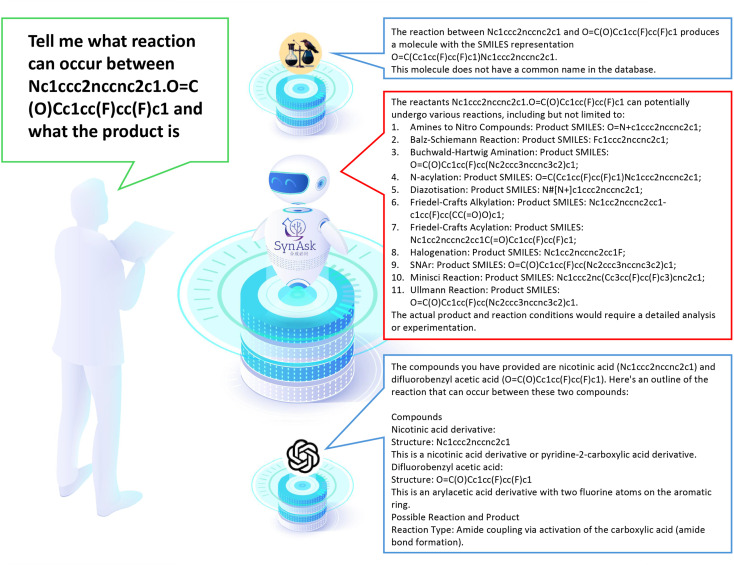
The comparison of SynAsk, ChatGPT-4, and ChemCrow output on a professional synthetic question.

As shown in Fig. 7, SynAsk demonstrates its specialization in organic chemistry by providing a comprehensive list of potential reactions and their corresponding products. Leveraging its domain-specific knowledge, SynAsk offers a diverse array of feasible transformations, including *N*-acylation, the Buchwald–Hartwig amination, and Minisci reaction, among others. This exhaustive output underscores SynAsk's capacity to analyze complex molecular interactions and propose multiple viable pathways.

In contrast, ChemCrow delivers a singular response, identifying the reaction as *N*-acylation and providing the corresponding product. While ChemCrow offers a concise solution, its limitation in providing alternative reaction pathways restricts its utility in scenarios where multiple transformation possibilities exist.

ChatGPT-4, although proficient in understanding the query, encounters a misinterpretation in identifying the compounds involved. While it accurately delineates the structure and classification of the provided molecules, it erroneously labels Nc1ccc2nccnc2c1 as a nicotinic acid derivative, instead of recognizing it as quinoxalin-6-amine. This discrepancy underscores the model's susceptibility to misinterpretation of chemical structures, particularly in complex contexts.

SynAsk distinguishes itself as a specialized LLM tailored specifically for organic chemistry tasks. Its domain-specific training and integration of fine-tuning techniques result in a robust model capable of providing detailed insights and accurate predictions for complex synthetic queries. While ChatGPT-4 and ChemCrow offer general language processing capabilities, they lack the nuanced understanding and domain expertise exhibited by SynAsk in the context of organic chemistry applications. Therefore, for researchers seeking nuanced insights and comprehensive analyses in organic synthesis, SynAsk stands as a valuable tool for augmenting chemical exploration and discovery.

### 3.4 SynAsk limitations

SynAsk, while robust, has some current limitations. Although it integrates many tools, it does not yet fully cover every aspect of organic chemistry that researchers may require. In terms of reaction types, SynAsk primarily addresses common medicinal chemistry reactions, but there are still gaps in its ability to classify all reaction types or predict yields for more complex cases. Additionally, some tools, particularly for retrosynthesis, can have slower response times, though users are notified in advance when longer processing is expected. Language support is also currently limited, as SynAsk has been developed and trained primarily in English and Chinese, with reduced functionality in other major scientific research languages. Nonetheless, these are areas of active development, and we continue to expand SynAsk's capabilities.

## 4 Conclusions and future work

In this work, we have developed SynAsk, a specialized LLM-powered platform for synthetic chemistry. It represents the first publicly accessible chemistry domain-specific LLM, fine-tuned with selected chemistry data and connected with both in-house and external cheminformatics tools. Through comparative analyses with foundation LLMs, we have demonstrated SynAsk's proficiency and specialization in synthetic chemistry. Results obtained in reaction yield prediction and retrosynthesis further validate SynAsk's capability in providing valuable chemical insights to synthetic chemists across multiple domains.

Looking ahead, our future endeavors aim to enhance the functionality of SynAsk by empowering the language model and fine-tuning it with additional data for more seamless and appropriate responses. Additionally, we envision SynAsk playing a pivotal role in driving autonomous reaction laboratories.^54^ Traditionally, reaction robots have been constrained by written scripts to define their scopes. Recent research has showcased the potential of LLMs to drive robotic chemists effectively.^55^ Leveraging SynAsk's capabilities such as retrosynthesis, inference, and programming script writing, we foresee it being instrumental in driving autonomous laboratories, representing the next phase of our fusion of LLM and hardware research.

## Author contributions

K. Liao conceived and supervised the project. C. Zhang and B. Zhu developed the model, while Q. Lin prepared the data. H. Yang, X. Lian, H. Deng, and J. Zheng conducted the evaluations. The manuscript was written with contributions from C. Zhang, Q. Lin, B. Zhu, and K. Liao. All authors approved the final version of the manuscript.

## Conflicts of interest

We have a patent application in China with the application number 202410714040.6 titled “A Human–Computer Interaction Method and Electronic Device Based on a Large Language Model”.

## Supplementary Material

SC-016-D4SC04757E-s001

## Data Availability

Due to legal and ethical confidentiality constraints, the data supporting the findings of this study are not publicly available. These restrictions prohibit the sharing of the data to protect the privacy and confidentiality of individuals involved in the research.
