# Supplementary material for: SynAsk: unleashing the power of large language models in organic synthesis
Source: Chem Sci. 2024 Nov 18;16(1):43–56. doi: 10.1039/d4sc04757e (PMC11587532; doi:10.1039/d4sc04757e)
Supplement: SC-016-D4SC04757E-s001 [file SC-016-D4SC04757E-s001.pdf]

Supplementary Information (SI) for Chemical Science.
This journal is © The Royal Society of Chemistry 2024

SynAsk: Unleashing the Power of Large Language Models in Organic Synthesis

Electronic Supplementary Information

Chonghuan Zhang[1†], Qianghua Lin[1†], Biwei Zhu[2†], Haopeng Yang[2], Xiao Lian[2], Hao Deng[2], Jiajun Zheng[2], Kuangbiao Liao[1*]

[1]Guangzhou National Laboratory, Guangdong, PR China, 510005
[2]AIChemEco Inc., Guangdong, PR China, 510005

[*] Corresponding author(s). E-mail(s): kuangbiao_liao@gzlab.ac.cn
[†]These authors contributed equally to this work.

S1 The indicators used to assess LLMs

We evaluated the LLM's capabilities using various metrics including Massive Multi-task Language Understanding (MMLU), Multi-level multi-discipline chinese evaluation (C-Eval), GSM8K, BIG-Bench-Hard (BBH), and Measuring massive multitask language understanding in Chinese (CMMLU). These metrics collectively provide a thorough assessment of a model's proficiency, encompassing linguistic understanding, mathematical reasoning, contextual comprehension, multimodal integration, and the application of CoT, which examines the fluency of LLMs' integration with external tools. This evaluation framework emphasizes the diverse and essential skills a model needs to effectively tackle complex real-world problems.

- **Massive Multi-task Language Understanding, MMLU** represents a comprehensive and multifaceted initiative that aims to evaluate and enhance the performance of language models across a broad range of linguistic challenges, providing an extensive evaluation of global knowledge and problem-solving abilities.
- **Multi-level Multi-discipline Chinese Evaluation, C-Eval** tests models in scenarios that necessitate an understanding of subtle context, which is crucial for applications involving natural language understanding and generation.
- **Grade School Math 8K, GSM8K** is a widely recognized test set designed to assess the mathematical capabilities of language models. It comprises problems that require 2-8 steps of basic mathematical operations to test the models' multi-step mathematical reasoning.
- **BIG-Bench-Hard, BBH** evaluates language models' capabilities in applying Chain of Thought to humanistic knowledge. It measures how effectively a model can navigate through complex humanistic concepts and ideas, emphasizing its ability to perform sequential reasoning that mirrors human-like understanding in tasks with cultural and historical depth.
- **Measuring massive multitask language understanding in Chinese, CMMLU** is a comprehensive Chinese evaluation benchmark specifically used to evaluate the knowledge and reasoning capabilities of language models in the Chinese context. CMMLU covers 67 topics from basic subjects to advanced professional levels.

Model	MMLU (5-shot)	C-Eval (5-shot)	GSM8K (8-shot)	BBH (3-shot)	CMMLU
LLaMA2-7B	46.8	32.5	16.7	38.2	31.8
LLaMA2-13B	55.0	41.4	29.6	45.6	38.4
LLaMA2-32B	62.6	-	42.2	44.1	-
ChatGLM2-6B	47.9	51.7	32.4	33.7	-
InterLM-7B	51.0	53.4	31.2	37.0	51.8
InterLM-20B	62.1	58.8	52.6	52.5	59.0
Baichuan2-7B	54.7	56.3	24.6	41.6	57.1
Baichuan2-13B	59.5	59.0	52.8	49.0	62.0
Yi-34B	76.3	81.8	67.9	66.4	85.6
Qwen-1.8B	45.3	56.1	32.3	22.3	52.1
Qwen-7B	58.2	63.5	51.7	45.0	62.2
Qwen-14B	66.3	72.1	61.3	53.4	71.0
Qwen-72B	77.4	83.3	78.9	67.7	83.6

Table S1: Performance of Different Models on Various Benchmarks

S2 Fine-tuning techniques and procedures

In our experiments, we explored two distinct fine-tuning methodologies for LLMs. The first approach involved techniques such as quantization to enable the operation of a 14-billion-parameter model within a 24GB GPU environment. The second approach was direct fine-tuning without additional quantization techniques.

For our experiments, we selected a model with 14 billion parameters. We applied Low-Rank Adaptation (LoRA) by incorporating low-rank matrices into the fully connected layers. The parameter details are presented in Table S2.

**Total Parameters**	**Trainable Parameters**	**Percentage of Total**
14,209,134,120	41,843,040	$\approx 0.294\%$

Table S2: Parameter quantity of the 14-billion-parameter model

The fine-tuning process, including quantization, was performed on a dataset of 200 entries with a batch size of 2 and completed within an hour. This serves as a reference for estimating the time required to fine-tune larger datasets under similar computational constraints. This method is a viable solution for managing large model training on hardware with limited memory without significantly compromising precision.

	**Before Quantization**	**After 4-bit Quantization**
During loading	$14 \times 10^9 \times 4$ bytes	$14 \times 10^9 \times 0.5$ bytes
During computation	$14 \times 10^9 \times 2$ bytes	
**Memory Consumption**	≈ 56 GB	≈ 7 GB during loading

Table S3: Memory usage before and after quantization

Leveraging a single GeForce RTX 4090 with 24GB of VRAM for fine-tuning a 14-billion-parameter model, we initially applied quantization to reduce the memory usage and accelerate inference, though at the potential cost of precision loss. During loading, the model was quantized to 4-bit precision and subsequently converted to 16-bit for computations. Post-loading, neither the original nor the quantized weights were retained in memory.

The fine-tuning without quantization approach utilized LoRA under the deepspeed's ZeRO-3 optimization. We employed three GeForce RTX 4090 GPUs, each with 24GB of memory, which allowed the fine-tuning of the model on a dataset of over 4,000 entries. The process took approximately seven hours to complete. This serves as a reference for estimating the time required to fine-tune larger datasets under similar computational constraints.

Both fine-tuning methodologies proved to be effective, demonstrating the practical applicability of our approaches to large-scale model optimization.

S3 Chemistry related indicators and examples

We assessed the chemistry ability of the LLMs using the chemistry test questions from C-Eval, which comprises multiple discipline questions in multiple levels in Chinese (Section S1). This test was completed in Chinese since SynAsk's original language is Chinese. However, we acknowledge that with the LLMs' powerful language ability, testing of the LLMs with different major languages in the world would reach close results.

We provide a set of example questions for the chemistry question in C-Eval at multiple levels. Sections Section S3.1, Section S3.2, and Section S3.3 refers to the chemistry questions at college, high school and middle school levels, respectively. The dataset format consists of multiple-choice questions and answers. The Prediction contains the answers predicted by three models: SynAsk, Qwen1.5-14B-Chat, and Qwen-14B-Chat.

S3.1 C-Eval (College Chemistry)

Problem:
以下是中国关于大学化学考试的单项选择题，请选出其中的正确答案。
下列说法中，正确的是：

(A) 单质的焓为零

(B) 反应的热效应就是该反应的摩尔焓变

(C) 单质的摩尔生成焓为零

(D) 由最稳定单质生成1 mol化合物时，该化合物的标准摩尔生成焓 $\Delta_{\mathfrak{f}} H_{\mathfrak{m}}^{\mathfrak{e}}$ 等于该生成反应的 $\Delta_{\mathfrak{r}} H_{\mathfrak{m}}^{\mathfrak{e}}$

English translation:
The following are single-choice questions on university chemistry exams in China. Which of the following statements is correct?

(A) The enthalpy of an element is zero

(B) The heat of reaction is equal to the molar enthalpy change of the reaction

(C) The molar enthalpy of formation of an element is zero

(D) When 1 mole of a compound is formed from the most stable elements, the standard molar enthalpy of formation $\Delta_{\mathfrak{f}} H_{\mathfrak{m}}^{\mathfrak{e}}$ of the compound is equal to the standard molar enthalpy of reaction $\Delta_{\mathfrak{r}} H_{\mathfrak{m}}^{\mathfrak{e}}$ of the formation reaction

Answer: D

Predictions:

SynAsk: D

Qwen1.5-14B-Chat: C

Qwen-14B-Chat: C

S3.2 C-Eval (High School Chemistry)

Problem:
以下是中国关于高中化学考试的单项选择题，请选出其中的正确答案。
下列说法中，正确的是：
在一定温度下的恒容密闭容器中，当下列哪些物理量不再发生变化时，表明下述反应：
$A(s) + 2B(g) \rightleftharpoons C(g) + D(g)$ 已达到平衡状态：
①混合气体的压强
②混合气体的密度
③B的物质的量浓度
④气体的总物质的量
⑤混合气体总质量

(A) ②③⑤

(B) ①②③

(C) ②③④

(D) ①③④⑤

English translation:
The following are single-choice questions on high school chemistry exams in China. Please select the correct answer.
Which of the following statements is correct?
In a constant-volume sealed container at a certain temperature, when which of the following physical quantities no longer change, it indicates that the following reaction:

$$A(s) + 2B(g) \rightleftharpoons C(g) + D(g)$$

has reached equilibrium:

① Pressure of the mixed gases

② Density of the mixed gases

③ Concentration of substance B

④ Total amount of gas

⑤ Total mass of the mixed gases

(A) ②③⑤

(B) ①②③

(C) ②③④

(D) ①③④⑤

Answer: A

Predictions:

SynAsk: A

Qwen1.5-14B-Chat: ②③⑤

Qwen-14B-Chat: A

It is noted while Qwen1.5-14B-Chat provides with the right answer, it predicts with the context of the answer directly without showing the correct choice “A".

S3.3 C-Eval (Middle School Chemistry)

Problem:
以下是中国关于初中化学考试的单项选择题，请选出其中的正确答案。
下列有关实验现象的描述正确的是：

(A) 硫在氧气中燃烧发出淡蓝色火焰

(B) 无色酚酞试液遇稀盐酸变成红色

(C) 硫酸铜溶液和氢氧化钠溶液反应会产生蓝色沉淀

(D) 红磷在空气中燃烧产生白雾

English translation:
The following are single-choice questions on junior high school chemistry exams in China. Please select the correct answer.
Which of the following descriptions about experimental phenomena is correct?

(A) Sulfur burns with a pale blue flame in oxygen

(B) Colorless phenolphthalein turns red when mixed with dilute hydrochloric acid

(C) The reaction between copper sulfate solution and sodium hydroxide solution produces a blue precipitate

(D) Red phosphorus burns in air to produce white smoke

Answer: C

Predictions:

SynAsk: C

Qwen1.5-14B-Chat: C

Qwen-14B-Chat: C

S4 Reaction yield prediction results

For the nucleophilic aromatic substitution (S_NAr) reaction case study, to address the generalizability of the HTE substrate sampling, we developed a novel method designed to mimic real-world scenarios and generalize to more complex reactions across a broader chemical landscape. This approach involves projecting literature and patented reactions into chemical space, followed by virtual compound generation, dimensionality reduction, clustering, virtual compound filtering, and stratified sampling. The selected substrates (77 amines and 64 halides) are shown in Figure S1.

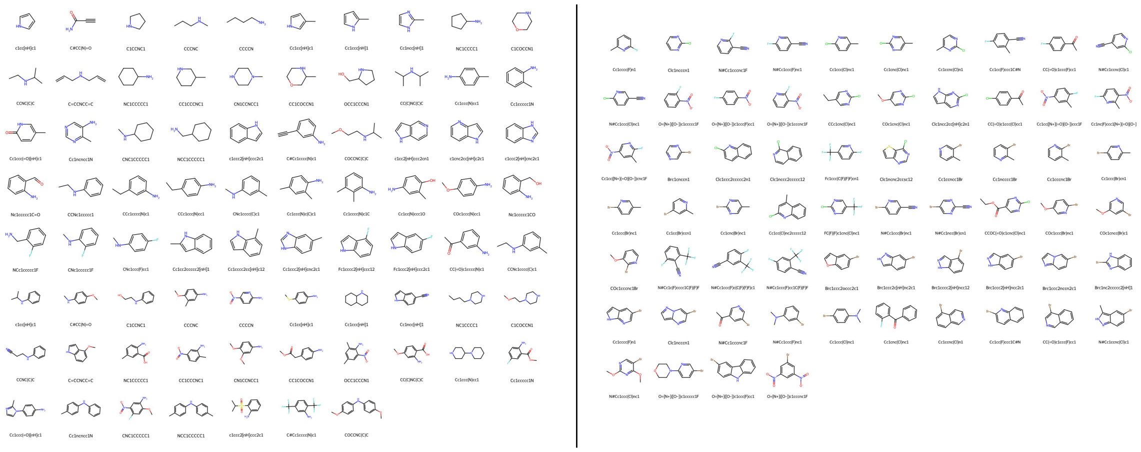

Figure S1: The 77 amines (left) and 64 halides (right) used for S_NAr reaction HTE substrate sampling.

We compared our experimental validations with the model predictions for test datasets across four major reaction models: SNAr, Suzuki cross-coupling, Buchwald-Hartwig cross-coupling, and amide coupling, as shown in Figure S2. These reaction models have been integrated into SynAsk as in-house reaction model tools. These plots also provide MSE, MAE, and R^2 values, further demonstrating the accuracy and reliability of our models across a diverse range of reaction types.

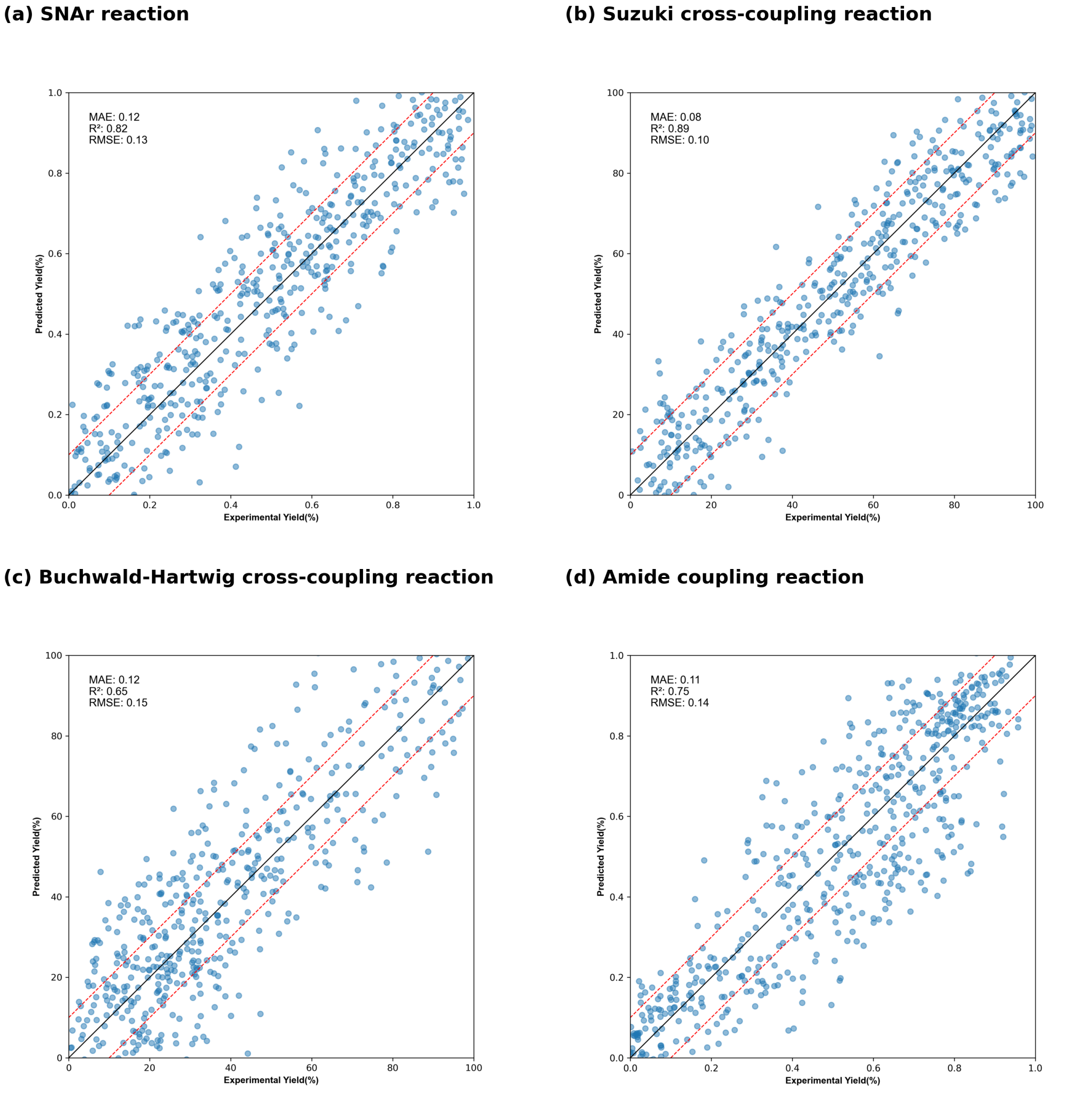

Figure S2: The experimental yield versus predicted yield for test datasets across four major reaction models embedded in SynAsk.

S5 Retrosynthetic pathway of selected target molecules

Figure S3, Figure S4, Figure S5 and Figure S6 shows numbers of retrosynthetic pathways generated by SynAsk, which provide insights for synthetic chemists. The routes indicate the ability of SynAsk in computer assisted synthetic planning (CASP).

We are developing strategies towards generation of more reasonable retrosynthetic pathways. This will be published elsewhere, and integrated into SynAsk. Till now, no efforts were made to experimentally validate the synthetic routes provided, and more synthetic routes to other target molecules can be generated via command to SynAsk.

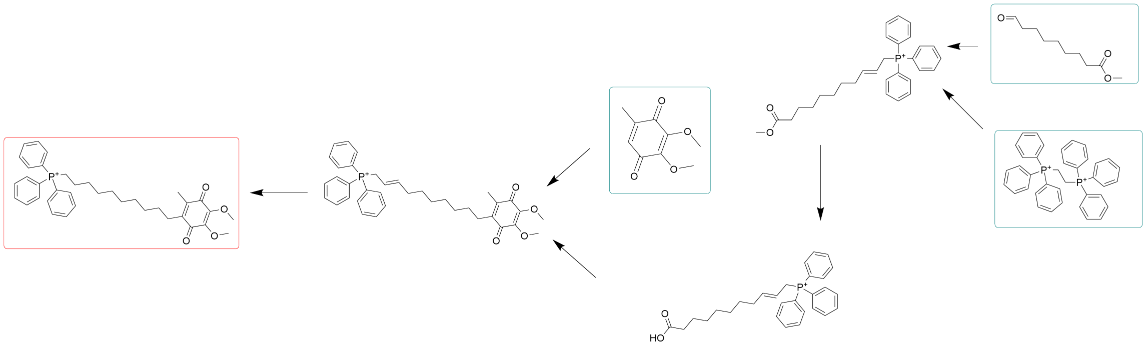

Figure S3: The synthetic route of the target molecule mitoquinone planned by SynAsk's retrosynthetic tool.

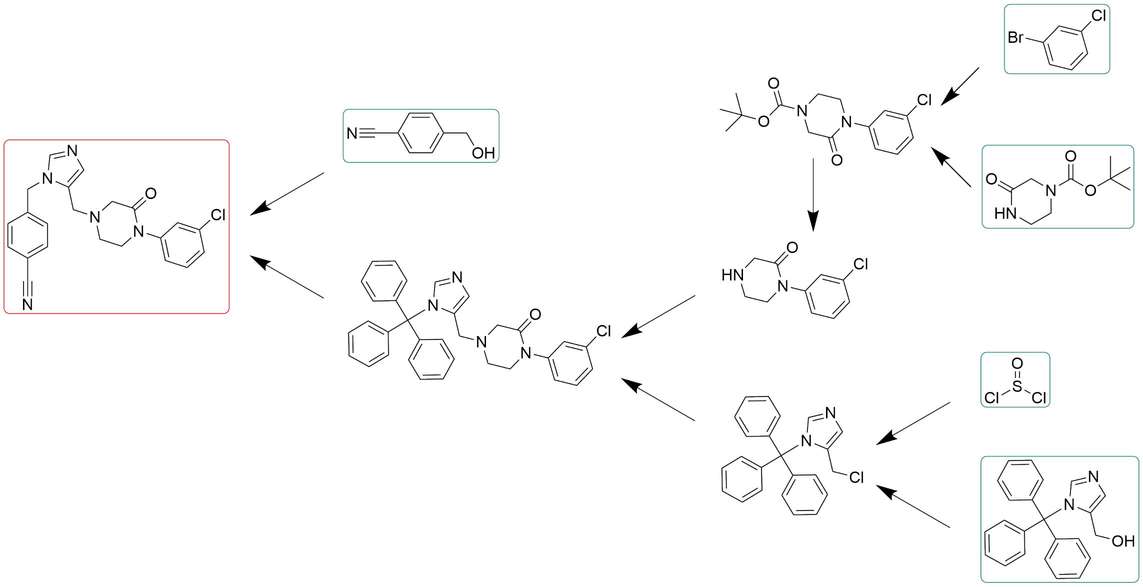

Figure S4: The synthetic route of the target molecule L-778123 planned by SynAsk's retrosynthetic tool.

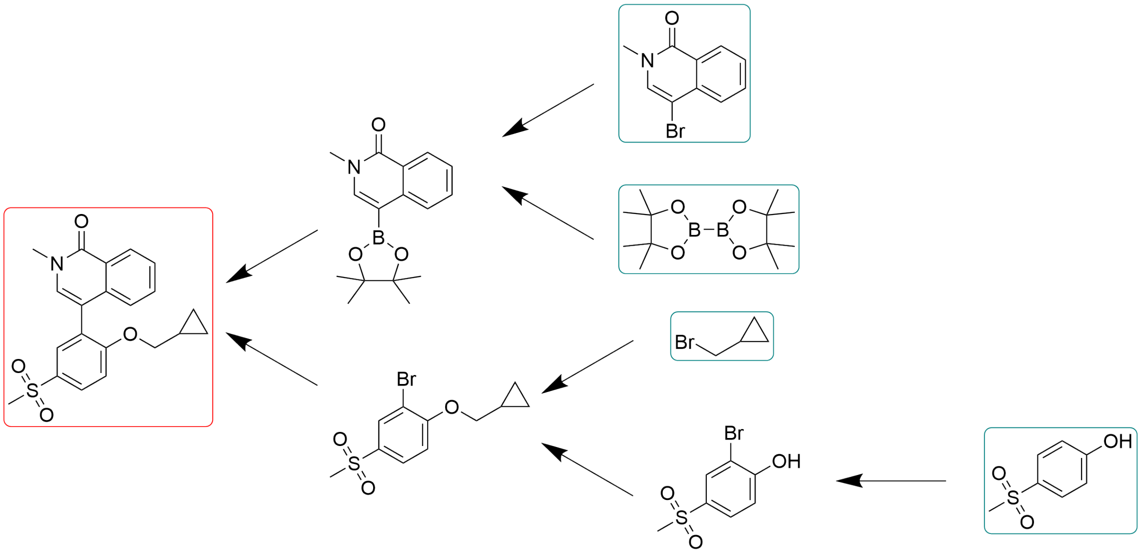

Figure S5: The synthetic route of the target molecule trotabresib planned by SynAsk's retrosynthetic tool.

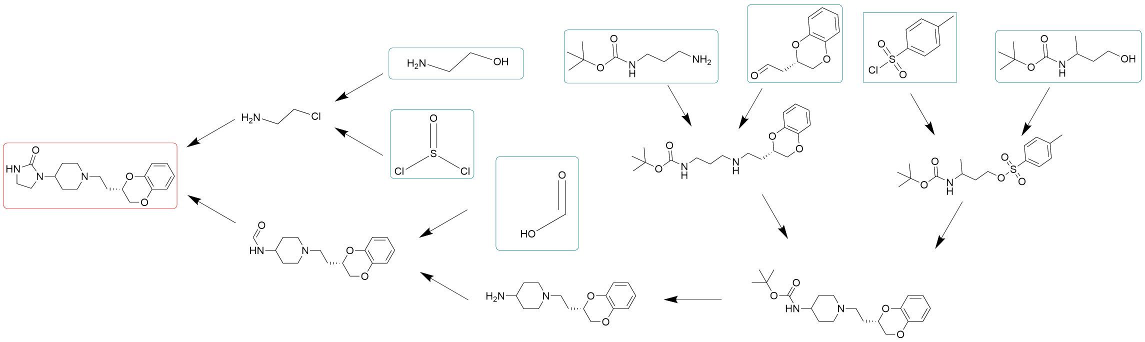

Figure S6: The synthetic route of the target molecule azaloxan planned by SynAsk's retrosynthetic tool.

S6 Examples of the SynAsk platform outputs versus other LLMs

User

what is dopamine?

SynAsk

Dopamine is a neurotransmitter, a chemical messenger, that plays a crucial role in the brain. It is primarily involved in the regulation of mood, motivation, reward, and movement. Dopamine is often associated with feelings of pleasure and reinforcement, and imbalances in dopamine levels have been linked to various neurological and psychological conditions, such as Parkinson's disease and addiction.

ChemCrow

Dopamine (DA, a contraction of 3,4-dihydroxyphenethylamine) is a neuromodulatory molecule that plays several important roles in cells. It is an organic chemical of the catecholamine and phenethylamine families. Dopamine constitutes about 80% of the catecholamine content in the brain. It is an amine synthesized by removing a carboxyl group from a molecule of its precursor chemical, L-DOPA, which is synthesized in the brain and kidneys. Dopamine is also synthesized in plants and most animals. In the brain, dopamine functions as a neurotransmitter—a chemical released by neurons (nerve cells) to send signals to other nerve cells. The brain includes several distinct dopamine pathways, one of which plays a major role in the motivational component of reward-motivated behavior. Most types of rewards increase the level of dopamine in the brain, and many addictive drugs increase dopamine release or block its reuptake into neurons following release. Other brain dopamine pathways are involved in motor control and in controlling the release of various hormones. These pathways and cell groups form a dopamine system which is neuromodulatory. Dopamine is often portrayed as the main chemical of pleasure, but the current opinion in pharmacology is that dopamine instead confers motivational salience. Outside the central nervous system, dopamine functions primarily as a local paracrine messenger. In blood vessels, it inhibits norepinephrine release and acts as a vasodilator; in the kidneys, it increases sodium excretion and urine output; in the pancreas, it reduces insulin production; in the digestive system, it reduces gastrointestinal motility and protects intestinal mucosa; and in the immune system, it reduces the activity of lymphocytes. Several important diseases of the nervous system are associated with dysfunctions of the dopamine system, and some of the key medications used to treat them work by altering the effects of dopamine. Parkinson's disease, a degenerative condition causing tremor and motor impairment, is caused by a loss of dopamine-secreting neurons in an area of the midbrain called the substantia nigra. Its metabolic precursor L-DOPA can be manufactured; Levodopa, a pure form of L-DOPA, is the most widely used treatment for Parkinson's. There is evidence that schizophrenia involves altered levels of dopamine activity, and most antipsychotic drugs used to treat this are dopamine antagonists which reduce dopamine activity. Similar dopamine antagonist drugs are also some of the most effective anti-nausea agents. Restless legs syndrome and attention deficit hyperactivity disorder (ADHD) are associated with decreased dopamine activity. Dopaminergic stimulants can be addictive in high doses, but some are used at lower doses to treat ADHD. Dopamine itself is available as a manufactured medication for intravenous injection. It is useful in the treatment of severe heart failure or cardiogenic shock. In newborn babies it may be [illegible]

ChatGPT-4

Dopamine is a neurotransmitter, a chemical messenger that plays a crucial role in transmitting signals in the brain and other areas of the body. Here are some key points about dopamine:

Chemical Properties
Chemical Structure: C8H11NO2
Systematic Name: 4-(2-aminoethyl)benzene-1,2-diol
Classification: Catecholamine and phenethylamine
Abbreviation: DA
Function in the Nervous System
Neurotransmitter Role:
Dopamine is primarily a neurotransmitter in the central nervous system (CNS). It is synthesized in dopaminergic neurons from the amino acid tyrosine via the intermediate L-DOPA.
Major Dopaminergic Pathways in the Brain:
Mesolimbic Pathway: Related to reward, pleasure, and reinforcement learning.
Mesocortical Pathway: Associated with cognition and executive function.
Nigrostriatal Pathway: Involved in movement regulation; degeneration of neurons in this pathway is linked to Parkinson's disease.
Tuberoinfundibular Pathway: Inhibits prolactin release from the pituitary gland.
Physiological Functions
Reward and Pleasure:
Dopamine is often called the "feel-good" neurotransmitter because it contributes to feelings of pleasure and reward.
Motor Control:
In the nigrostriatal pathway, dopamine helps regulate movement. Its deficiency leads to motor symptoms seen in Parkinson's disease.
Cognition and Attention:
It plays a role in memory, attention, and problem-solving.
Hormone Regulation:
Inhibits prolactin release from the anterior pituitary.
Clinical Significance
Neurological Disorders:
Parkinson's Disease: Characterized by a loss of dopamine-producing neurons in the substantia nigra.
Schizophrenia: Linked to dysregulated dopamine activity, especially in the mesolimbic pathway.
Psychiatric Disorders:
Depression: Dopamine dysregulation is implicated in certain depressive disorders.
ADHD (Attention Deficit Hyperactivity Disorder): Related to dopamine function in the prefrontal cortex.
Addiction:
Dopamine release is associated with addictive behaviors due to its role in the reward pathway.
Synthesis and Metabolism
Biosynthesis:
Precursor: Tyrosine
Intermediate: L-DOPA
Enzymes: Tyrosine hydroxylase (tyrosine to L-DOPA) and DOPA decarboxylase (L-DOPA to dopamine)
Catabolism:
Metabolized by monoamine oxidase (MAO) and catechol-O-methyltransferase (COMT) to homovanillic acid (HVA), which is excreted in the urine.
Applications and Treatments
Pharmacology:
Levodopa (L-DOPA): Used to treat Parkinson's disease.
Dopamine Agonists: Drugs that mimic dopamine effects in the brain.
Dopamine Antagonists: Block dopamine receptors and are used in treating schizophrenia.
Clinical Monitoring:
Dopamine levels can be assessed through homovanillic acid (HVA) levels in the cerebrospinal fluid or urine.
If you need further elaboration on any specific aspect or its role in particular disorders, I'd be happy to expand on that!

Figure S7: The first example of the outputs from the LLMs.

User

Can you recommend me some reaction conditions for Suzuki cross-coupling?

SynAsk

The recommended reaction conditions for Suzuki cross-coupling are as follows:
Condition 1: Reagents: Pd(PPh3)4 (0.05 equiv); Reagents: K2CO3 (3.0 equiv); Solvent: THF/H2O (4:1); Reaction conditions: 100°C, 12h
Condition 2: Reagents: PdCl2(PPh3)2 (0.05 equiv); Reagents: Na2CO3 (3.0 equiv); Solvent: THF/H2O (4:1); Reaction conditions: 100°C, 12h
Condition 3: Reagents: Pd(OAc)2 (0.05 equiv); Reagents: dppf (0.1 equiv); Reagents: Cs2CO3 (3.0 equiv); Reagents: CuCl (1.0 equiv); Solvent: DMF; Reaction conditions: 100°C, 12h

ChemCrow

The Suzuki-Miyaura cross-coupling reaction is a pivotal method in organic chemistry for the formation of carbon-carbon bonds. The efficiency of this reaction hinges on the careful selection of reaction conditions, such as the choice of solvent, catalyst, and particularly, the reaction temperature, to ensure the desired coupling while minimizing side reactions. For instance, in the optimization of conditions for diazirine-containing compounds, a temperature of 40°C was identified as optimal. This temperature not only preserved the diazirine moiety but also limited its participation in competitive side reactions. The choice of coupling partners is also crucial, with bromoquinoline showing higher conversion efficiency than chloroquinoline. This specificity underscores the importance of selecting appropriate reaction parameters to achieve efficient coupling, which is essential for the synthesis of pharmaceutically relevant structures and the broader application of the Suzuki-Miyaura reaction in organic synthesis and material science.

ChatGPT-4

The Suzuki cross-coupling reaction, or Suzuki-Miyaura coupling, is a powerful method for forming carbon-carbon bonds between organoboranes (typically boronic acids) and organohalides or pseudohalides in the presence of a palladium catalyst. Here are some common reaction conditions for this versatile reaction:

General Reaction Scheme
R–X + R'–B(OR)2 + Base + Pd catalyst ⟶ R–R'

Common Reaction Components
Substrates:
R–X: Aryl or vinyl halides (X = I, Br, Cl) or pseudohalides (OTf, N2, etc.)
R'–B(OR)2: Boronic acids, boronate esters, trifluoroborates, etc.
Catalysts:
Pd(PPh3)4
Pd(OAc)2/PPh3
Pd2(dba)3/P(tBu)3
PdCl2(dppf)
Bases:
Inorganic: K2CO3, Cs2CO3, Na2CO3, NaHCO3, NaOH
Organic: Et3N, iPr2NEt, DBU
Solvents:
Aqueous Organic: DMF/H2O, THF/H2O, DME/H2O
Non-Aqueous: Toluene, dioxane, DME, MeOH, DMF
Additives:
Ligands: Phosphines like PPh3, P(tBu)3, PCy3, BINAP, SPhos, XPhos
Stabilizers: Tetrabutylammonium salts
Example Conditions
1. Standard Conditions
Aryl Halide: R–Br or R–I
Boronic Acid: R'–B(OH)2
Catalyst: Pd(PPh3)4 (1-5 mol%)
Base: K2CO3 or Cs2CO3
Solvent: Toluene/H2O (4:1), DME/H2O, or EtOH/H2O
Temperature: 80–100 °C
Time: 12–24 hours
Example Reaction Setup:

Aryl Halide (1 mmol)
Boronic Acid (1.2 mmol)
Pd(PPh3)4 (3 mol%)
K2CO3 (2 mmol)
Toluene/H2O (4:1, 5 mL)
80 °C, 12 h
2. Chloride Activation
Aryl Halide: R–Cl
Boronic Acid: R'–B(OH)2
Catalyst: PdCl2(dppf) or Pd2(dba)3/SPhos
Base: K3PO4 or CsF
Solvent: 1,4-Dioxane or DMF
Temperature: 80–120 °C
Example Reaction Setup:

Aryl Chloride (1 mmol)
Boronic Acid (1.2 mmol)
PdCl2(dppf) (3 mol%)
K3PO4 (2 mmol)
1,4-Dioxane (5 mL)
100 °C, 24 h
3. Water-Based Conditions
Aryl Halide: R–Br
Boronic Acid: R'–B(OH)2
Catalyst: Pd(OAc)2/SPhos or Pd(OAc)2/PPh3
Base: K2CO3, NaOH
Solvent: H2O or H2O/EtOH (1:1)
Example Reaction Setup:

Aryl Bromide (1 mmol)
Boronic Acid (1.2 mmol)
Pd(OAc)2 (2 mol%)
SPhos (4 mol%)
NaOH (2 mmol)
H2O/EtOH (1:1, 5 mL)
80 °C, 12 h
Notes on Optimizing Conditions
Ligand Choice: Bulky phosphine ligands like SPhos and XPhos work well with less reactive aryl chlorides.
Base Selection: Inorganic bases like K2CO3 and Na2CO3 are commonly used, but stronger bases (NaOH, CsF) may be required for less reactive halides.
Temperature: Typically 80–120 °C; adjust for substrate reactivity.
Catalyst Loading: 1–5 mol% is common; higher loading may be needed for sterically hindered substrates.
These guidelines provide a starting point for experimenting with Suzuki coupling reactions. Do you need specific conditions tailored to a particular set of substrates?

Figure S8: The second example of the outputs from the LLMs.

User

Please help me find some literature related to C-H activation

SynAsk

Here are some relevant articles on C-H activation:

1. "A Prodigious Behavior of Cycloalkyl Carboxylic Acid to Access 2D Space from 3D Space via Multifold CH Activation" by D. Maiti, T. Pal, et al. (2023) - Describes multifold CH activation in carboxylic acids for organic synthesis.
2. "Pyridine-Based 1, 2, 3-Triazoles: A New Class of Potential KAT2A Inhibitors and Scaffolds for CH Activation and Catalysis" by R. Pacifico (2023) - Discusses the use of pyridine-based triazoles in CH activation and their potential applications.
3. "The catalytic role of imido and amido complexes in the ruthenium porphyrins catalyzed allylic CH activation" by E. Gallo, S. Fantauzzi, et al. (2008) - Investigates the role of ruthenium porphyrins in allylic CH activation.

These articles provide insights into the mechanisms, catalysts, and potential applications of C-H activation in various chemical processes. For more detailed information, you can access the full articles through the provided links.

ChemCrow

Here are some literature related to C-H activation:

1. Liangliang Song and E. V. Van der Eycken. "Transition metal-catalyzed intermolecular cascade c-h activation/annulation processes for the synthesis of polycycles." Chemistry, 2020. This paper discusses significant advancements in the field of C-H activation, particularly in the synthesis of polyheteroaromatic compounds and other complex molecules through innovative strategies such as rhodium(III)-catalyzed double C-H activation/annulation reactions.
2. Richmond Sarpong. "C–h functionalization/activation in organic synthesis." Beilstein Journal of Organic Chemistry, 12:2315 - 2316, 2016. This paper discusses the growth in popularity of C-H functionalization/activation due to its potential for more sustainable synthesis methods and the pursuit of novel reactivity and selectivity in molecule building.

ChatGPT-4

C-H activation is a significant area of research in organic chemistry that involves the activation of otherwise inert C-H bonds to facilitate their functionalization. Here are some sources and directions to guide your literature search:

General Overview
Review Articles:
Yu, J.-Q.; Shi, Z. (Eds.) "C-H Activation." Springer, 2010.
Labinger, J. A.; Bercaw, J. E. "Understanding and exploiting C-H bond activation." Nature, 2002, 417(6888), 507-514.
Books:
Crabtree, R. H. "The Organometallic Chemistry of the Transition Metals." Wiley, 2014.
Yamaguchi, J.; Yamaguchi, A. D.; Itami, K. "C-H Bond Functionalization: Emerging Synthetic Tools." Angew. Chem. Int. Ed., 2012, 51(36), 8960-9009.
Specific Topics in C-H Activation
Transition Metal Catalysts:
Ritter, T.; Stahl, S. S. "Catalytic C-H Functionalization via C-H Bond Activation." Catalytic C-H Functionalization, Wiley-VCH, 2013.
Giri, R.; Shi, B. F.; Engle, K. M.; Maugel, N.; Yu, J.-Q. "Transition Metal-Catalyzed C-H Functionalization Reactions: Diastereoselectivity and Enantioselectivity." Chem. Soc. Rev., 2009, 38, 3242-3272.
Direct Arylation:
Seregin, I. V.; Gevorgyan, V. "Direct Transition Metal-Catalyzed Functionalization of Heteroaromatic Compounds." Chem. Soc. Rev., 2007, 36, 1173-1193.
Ackermann, L. "Carboxylate-Assisted Transition-Metal-Catalyzed C-H Bond Functionalization: Mechanism and Scope." Chem. Rev., 2011, 111(3), 1315-1345.
C-H Activation in Pharmaceuticals:
Li, B.-J.; Shi, Z.-J. "Challenging C-H Functionalization Reactions for Organic Synthesis." Chem. Soc. Rev., 2012, 41(15), 5588-5598.
Godula, K.; Sames, D. "C-H Bond Functionalization in Complex Organic Synthesis." Science, 2006, 312(5770), 67-72.
Recent Advances and Novel Methodologies
Recent Reviews:
Chen, Z.; Rong, M.-Y.; Nie, J.; Zhu, X.-F.; Shi, B.-F.; Ma, J.-A. "Catalytic Alkylation of Unactivated C(sp3)-H and C(sp2)-H Bonds via Radical Process." Chem. Soc. Rev., 2019, 48, 4921-4942.
Hartwig, J. F.; Larsen, M. A. "Undirected, Homogeneous C-H Bond Functionalization: Challenges and Opportunities." ACS Cent. Sci., 2016, 2(5), 281-292.
Cross-Coupling Reactions:
Sun, C.-L.; Li, B.-J.; Shi, Z.-J. "Direct C-H Transformation via Iron Catalysis." Chem. Rev., 2011, 111(3), 1293-1314.
Wencel-Delord, J.; Dröge, T.; Liu, F.; Glorius, F. "Towards Mild Metal-Catalyzed C-H Bond Activation." Chem. Soc. Rev., 2011, 40(9), 4740-4761.
Photo and Electrochemistry:
Cui, L.; Ackermann, L. "Electrocatalysis: C-H Activation via Anodic Oxidation." ACS Catal., 2021, 11(23), 14022-14029.
Liu, Z.; Chen, X.; Tan, J. D.; Wu, Y. Z.; Hartwig, J. F.; Zhao, P. "Visible-Light Photoredox-Catalyzed C-H Bond Functionalization with Metal Catalysts." Acc. Chem. Res., 2020, 53(6), 1314-1325.
Practical Search Strategies
Use Relevant Databases:
SciFinder: The most comprehensive database for chemical literature.
Web of Science: Provides citation connections between articles.
Google Scholar: Free and useful for initial searches.
Target Keywords:
"C-H Activation," "C-H Functionalization," "Transition Metal-Catalyzed," "Cross-Coupling," "Direct Arylation," etc.

Figure S9: The third example of the outputs from the LLMs.
